# Biofilm Formation and Antimicrobial Resistance of *Staphylococcus aureus* and *Streptococcus uberis* Isolates from Bovine Mastitis

**DOI:** 10.3390/vetsci11040170

**Published:** 2024-04-10

**Authors:** Carlos E. Fidelis, Alessandra M. Orsi, Gustavo Freu, Juliano L. Gonçalves, Marcos V. dos Santos

**Affiliations:** 1 Department of Animal Nutrition and Production, School of Veterinary Medicine and Animal Science, University of São Paulo (USP), Pirassununga 13635-900, Brazil; carlosfidelis@usp.br (C.E.F.); alessandramodenaorsi@gmail.com (A.M.O.); gustavofreu@usp.br (G.F.); 2 Department of Large Animal Clinical Sciences, College of Veterinary Medicine, Michigan State University, East Lansing, MI 48864, USA; goncal25@msu.edu

**Keywords:** antimicrobial resistance, biofilm producer ability, bovine mastitis, antimicrobial agents

## Abstract

**Simple Summary:**

*Staphylococcus* (*Staph.*) *aureus* and *Streptococcus* (*Strep.*) *uberis* are key causes of intra-mammary infection in dairy cows, and their ability to form biofilms is recognized as a significant virulence factor influencing mastitis pathogenesis and the response to antimicrobial treatment. This study aimed to evaluate (a) the biofilm producer ability and antimicrobial resistance of *Staph. aureus* (*n* = 197) and *Strep. uberis* (*n* = 119) isolated from cows with clinical and subclinical mastitis, and (b) the association between biofilm formation and antimicrobial resistance. Both *Staph. aureus* and *Strep. uberis* exhibited high biofilm formation ability. However, no correlation was found between the form of mastitis presentation (clinical or subclinical) and the biofilm-forming capacity. Moreover, a significant proportion of *Staph. aureus* and *Strep. uberis* isolates demonstrated resistance to penicillin, ampicillin, and tetracycline. Interestingly, we observed no association between biofilm formation ability and antimicrobial resistance.

**Abstract:**

This study aimed to assess (a) the biofilm producer ability and antimicrobial resistance profiles of *Staphylococcus* (*Staph.*) *aureus* and *Streptococcus* (*Strep.*) *uberis* isolated from cows with clinical mastitis (CM) and subclinical mastitis (SCM), and (b) the association between biofilm producer ability and antimicrobial resistance. We isolated a total of 197 *Staph. aureus* strains (SCM = 111, CM = 86) and 119 *Strep. uberis* strains (SCM = 15, CM = 104) from milk samples obtained from 316 cows distributed in 24 dairy herds. Biofilm-forming ability was assessed using the microplate method, while antimicrobial susceptibility was determined using the disk diffusion method against 13 antimicrobials. Among the isolates examined, 57.3% of *Staph. aureus* and 53.8% of *Strep. uberis* exhibited the ability to produce biofilm, which was categorized as strong, moderate, or weak. In terms of antimicrobial susceptibility, *Staph. aureus* isolates displayed resistance to penicillin (92.9%), ampicillin (50.8%), and tetracycline (52.7%). Conversely, *Strep. uberis* isolates exhibited resistance to penicillin (80.6%), oxacillin (80.6%), and tetracycline (37.8%). However, no significant correlation was found between antimicrobial resistance patterns and biofilm formation ability among the isolates.

## 1. Introduction

Bovine mastitis is one of the prevailing diseases of dairy cows globally, leading to significant economic losses in dairy herds and the dairy industry [1]. Among the major causative agents, *Staphylococcus aureus* and *Streptococcus uberis* have been described as major causes of mastitis [2], and their transmission mechanisms, genetic diversity, virulence factors, and antimicrobial resistance profile have been extensively studied [3].

*Staph. aureus* is recognized as a contagious pathogen associated with chronic intra-mammary infections (IMI) [4]. This pathogen poses a challenge for antibiotic therapy owing to its antimicrobial resistance characteristics, including its ability to survive within phagocytes and form biofilms [5,6]. Conversely, molecular studies showed that *Strep. uberis*, initially considered an environmental reservoir pathogen, has also the potential of contagious transmission [7,8]. Similarly, to *Staph. aureus*, managing *Strep. uberis* IMI proves challenging, with low cure rates [9]. This difficulty may be attributed, in part, to virulence factors of *Strep. uberis*, including its biofilm-forming capability [10].

Biofilm, which is characterized by an extracellular polysaccharide matrix, serves as a protective layer for microorganisms, allowing their proliferation within and subsequent release into the environment [11]. The initiation of biofilm formation starts with a small number of bacterial cells adhering to a substrate. Subsequently, these bacteria release an extracellular polymeric substance (EPS), which, in conjunction with host components, constructs the extracellular matrix. While primarily composed of polysaccharides, proteins, nucleic acids, and lipids, the structure and composition of biofilms exhibit significant variability [12]. Biofilms have effects on the public health and industrial considerations pertaining to their influence on the economy, energy utilization, equipment deterioration and the occurrence of infections [13].

Bacteria within biofilms exhibit enhanced survival in adverse environments and innate resistance to antibiotics, disinfectants, and host defense mechanisms [14]. This high antimicrobial resistance can be attributed to a modified chemical microenvironment, spore formation, reduced growth rate, antibiotic inactivation by the extracellular matrix, and the occurrence of horizontal gene transfer [11]. In additional, biofilm plays a significant role in inefficient wound healing and contributes to the persistence of chronic wounds [15]. Thus, biofilm production may significantly impact disease progression and treatment outcomes and may contribute to the proliferation of antimicrobial resistance [16].

The escalation of antimicrobial resistance (AMR) is marked by the emergence and global spread of novel resistance mechanisms. With the diminishing efficacy of antibiotics, specific infections are becoming increasingly challenging, and in some cases, impossible to treat. Public health concerns are, therefore, increasing with the AMR growing challenge [17].

While antimicrobial treatment remains a primary strategy for mastitis treatment in dairy cows, excessive antibiotic use may escalate antibiotic resistance [18,19,20]. Bacterial resistance not only undermines the efficacy of current therapies but also amplifies cross-resistance to antimicrobials used in both veterinary and human medicine [21]. *Staphylococcus* spp. isolated from bovine mastitis have been reported as developing resistance to multiple antimicrobial classes, including β-lactams, tetracyclines, aminoglycosides, amphenicols, macrolides, trimethoprim, lipopeptides, and lincosamides [17]. For *Strep. uberis*, AMR are linked mainly to gentamicin and tetracycline [22]. Consequently, the dynamic nature of antimicrobial resistance necessitates ongoing vigilance and monitoring.

Studies evaluating the relationship between biofilm production and antimicrobial resistance can offer deeper insights into the mechanisms that can influence the efficacy of antimicrobial therapy against *Staph. aureus* and *Strep. uberis* in dairy herds. Thus, this study aimed to evaluate (a) the biofilm production and antimicrobial resistance of *Staph. aureus* and *Strep. uberis* isolates from cows with clinical mastitis (CM) and subclinical mastitis (SCM), and (b) the potential association between biofilm production and antimicrobial resistance.

## 2. Materials and Methods

### 2.1. Staph. aureus and Strep. uberis Isolates

A total of 197 *Staph. aureus* (SCM = 111, CM = 86) and 119 *Strep. uberis* (SCM = 15, CM = 104) isolates were randomly selected from bovine milk samples obtained from 24 dairy farms from January 2015 to September 2016, and submitted to the Milk Quality Research Laboratory (Qualileite Lab) at the University of São Paulo, Brazil. SCM categorization included cows with somatic cell count (SCC) > 200,000 cells/mL or positive California mastitis test (CMT). CM was designated when cows exhibited visible milk changes, regardless of associated systemic inflammation signs [20]. Composite samples were collected from cows with SCM, while samples from affected mammary quarters were taken for CM cases. CM severity data were unavailable. Milk collection was performed according to the guidelines outlined by the National Mastitis Council [20].

*Staph. aureus* identification relied on colony morphology, Gram-positive staining, positive catalase and tube coagulase tests, and a positive latex agglutination test. *Strep. uberis* identification was based on Gram-positive staining, a negative catalase reaction, Christie—Atkins—Munch-Petersen (CAMP) test negativity (or positivity) along with esculin hydrolysis, and no reaction in the bile esculin test [23]. Additionally, all isolates were identified at the species level using Matrix-Assisted Laser Desorption Ionization–Time of Flight Mass Spectrometry (MALDI-TOF MS) with scores > 2.0 [24]. The isolates were cryopreserved at −80 °C in sterile tubes containing brain heart infusion broth (BBL—Becton Dickinson and Co., Le Point de Claix, France) supplemented with 10% glycerin until further analysis.

### 2.2. Biofilm Formation

Prior to assessing biofilm formation ability, preserved isolates were thawed and streaked onto blood agar plates (BBL—Becton Dickinson and Co., Le Point de Claix, France) supplemented with 5% bovine blood to confirm colony purity. Biofilm formation by *Staph. aureus* and *Strep. uberis* isolates followed Stepanovic et al. [25] methodology. Briefly, a single colony was reinoculated onto trypticase soy broth (TSB; BD, Sparks, MD, USA) and incubated at 37 °C for 24 h. Subsequently, bacterial suspensions standardized to a 0.5 McFarland standard were prepared using a DEN-1 McFarland densitometer (Bio-san, Riga, Latvia).

Next, 200 μL aliquots of each suspension were transferred in triplicate to 96-well flat-bottomed sterile polystyrene microplates and incubated at 37 °C ± 1 °C for 24 h. Following incubation, microplates were agitated, fixed, stained with crystal violet for 5 min, dried, and resolubilized using 33% (*v*/*v*) glacial acetic acid. Biofilm production was quantified using a microtiter-plate reader (Exert Plus UV, Asys Hitech, Seekirchen am Wallersee, Austria) set at 540 nm for *Staph. aureus* and 620 nm for *Strep. uberis*. The average OD value of the triplicate was compared with the OD value of the negative control (ODNC) to determine the isolate’s capacity to produce biofilm. The negative control was formed only sterile TSB. [25]. Each batch incorporated *Staph. epidermidis* ATCC 12228 (non-producing biofilm) and *Staph. epidermidis* ATCC 35984 (positive producing biofilm) for biofilm production, ensuring quality control.

### 2.3. Antimicrobial Susceptibility Testing

*Staph. aureus* and *Strep. uberis* antimicrobial susceptibility was determined using the disk diffusion method, as described by the Clinical and Laboratory Standard Institute (CLSI, 2021). To perform disk diffusion in agar, one to three colonies of each isolate were transferred using a platinum loop, from blood agar into tubes containing 5mL of sterile saline and homogenized. The solution was standardized at 0.5 McFarland (~10^8^ CFU/mL) by turbidimeter (Uniscience, São Paulo, Brazil) and subsequently inoculated homogeneously over the entire surface of Mueller Hinton Agar (MHA) medium plates using sterile cotton swabs. Then, the discs containing the antimicrobials were applied to the MHA plates, which were incubated at 35 °C for 18 h. Thirteen antimicrobials were evaluated: tetracycline (30 μg), ceftiofur (30 µg), oxacillin (1 µg), pirlimycin (2 µg), ampicillin (10 µg), enrofloxacin (5 µg), gentamycin (10 µg), cephalothin (30 µg), amoxicillin/clavulanic acid (30 µg), penicillin/novobiocin (40 µg), and erythromycin (15 µg). *Staph. aureus* (ATCC 29213) served as a quality-control strain.

Inhibition zone diameters were measured in millimeters, with isolates categorized as susceptible, intermediate, or resistant in accordance with CLSI [26] guidelines. Intermediate isolates were classified as resistant. Antimicrobials were grouped into eight classes: beta-lactams, cephalosporins, aminoglycosides, macrolides, fluoroquinolones, lincosamides, tetracyclines, and penicillin/novobiocin. Isolates resistant to three or more antimicrobial classes were labeled as multidrug-resistant (MDR) bacteria [27].

### 2.4. Data Analysis

Isolates were assessed for their biofilm-forming capacity, measured using OD values in comparison to the negative control strain (ODNC). A four-grade scale categorized the strains’ biofilm formation ability: non-producing biofilm (NP) (OD < ODNC), weak (ODNC < OD ≤ 2 × ODNC), moderate (2 × ODNC < OD ≤ 4 × ODNC), or strong (OD > 4 × ODNC) [25].

The FREQ procedure of SAS 9.4 (SAS Institute Inc., Cary, NC, USA) determined isolate frequencies concerning both biofilm formation and antimicrobial susceptibility. Evaluations focused on the distributions of isolates within the pathogen (*Staph. aureus* or *Strep. uberis*) and mastitis presentation form (CM or SCM).

A logistic regression model assessed the impact of biofilm production on antimicrobial resistance in *Staph. aureus* and *Strep. uberis* isolated from CM and SCM:

logit(pi) = β_0_ + β_1_ × biofilm + β_2_ × pathogen + β_3_ × mastitis + β_4_ × (biofilm × pathogen) + β_5_ × (biofilm × mastitis) + herd(random) + *e*,

In the equation, logit(pi) represents the probability of an isolate displaying resistance or susceptibility to a tested antimicrobial; β_0_ denotes the intercept; β_1_ stands for the regression coefficient indicating the effect of biofilm production ability (yes or no); β_2_ represents the regression coefficient for the effect of bacterial species (*Staph. aureus* or *Strep. uberis*); β_3_ signifies the regression coefficient for the effect of the mastitis presentation form (CM or SCM); β_4_ accounts for the regression coefficient representing the interaction between biofilm production ability and bacterial species; β_5_ represents the regression coefficient for the interaction between biofilm production ability and mastitis presentation form. ‘*e*’ denotes the random residual error. The model included ‘herd’ as a random effect. Analyses were conducted using PROC GLIMMIX of SAS (version 9.4; SAS Institute Inc., Cary, NC, USA). Statistical significance was declared when *p* < 0.05.

## 3. Results

### 3.1. Biofilm Formation Ability

The biofilm production ability of *Staph. aureus* and *Strep. uberis* isolates is summarized in Table 1. Among the 197 *Staph. aureus* isolates, 57.4% (113/197) displayed the capability to form biofilm. Similarly, 53.8% (64/119) of *Strep. uberis* isolates exhibited biofilm-producing ability.

For *S. aureus* isolates from CM, 45.3% (39/86) were classified as NP, 14.0% (12/86) as weak, 12.8% (11/86) as moderate, and 27.9% (24/86) as strong biofilm producers. Among *Staph. aureus* isolates from SCM, 41.0% (45/111) were classified as NP, 19.0% (21/111) as weak, 23.0% (26/111) as moderate, and 17.0% (19/111) as strong biofilm producers (Table 1). For *Strep. uberis* isolates from CM, 39.0% (41/104) were classified as NP, 13.0% (13/104) as weak, 29.0% (30/104) as moderate, and 19.0% (20/104) as strong biofilm producers. In the case of *Strep. uberis* isolates from SCM, 93.0% (14/15) were classified as NP, with a single isolate (7.0%) categorized as a strong biofilm producer.

### 3.2. Antimicrobial Activity

In vitro testing revealed high antimicrobial susceptibility among *Staph. aureus* isolates, with rates exceeding 90% for amoxicillin/clavulanic acid, oxacillin, cephalothin, gentamicin, enrofloxacin, pirlimycin, erythromycin, and penicillin/novobiocin (refer to Table 2). However, susceptibility percentages dropped to 47.2% for tetracycline, 49.2% for ampicillin, and notably, only 7.5% for penicillin. A total of 90 isolates (45.7%) exhibited resistance to both ampicillin and penicillin.

The in vitro susceptibility of *Strep. uberis* isolates exhibited high rates, exceeding 90% for amoxicillin/clavulanic acid, cephalothin, ceftiofur, gentamicin, and penicillin/novobiocin. However, notably lower susceptibility frequencies were observed for oxacillin (13.4%) and penicillin (12.6%).

Table 3 presents the outcomes of the generalized mixed model, assessing the impact of biofilm formation on resistance and multidrug resistance in *Staph. aureus* and *Strep. uberis* isolates. A statistical difference was observed between multiresistant Staph. aureus and *Strep. uberis* isolates (*p* = 0.016; Table 3), demonstrating that *Strep. uberis* isolates exhibited a higher likelihood of multidrug resistance in comparison to *Staph. aureus* isolates.

An individual analysis of antimicrobials revealed significant effects of the presentation form of mastitis (CM or SCM) and resistance to ampicillin. Specifically, SCM isolates exhibited a 2.7-fold higher likelihood of resistance compared to CM isolates. An interaction effect was observed solely between biofilm production ability and the presentation form of mastitis concerning resistance to gentamicin. However, no significant difference was observed between mastitis presentation form and biofilm production ability in the other antimicrobials.

The frequencies of *Staph. aureus* and *Strep. uberis* isolates were categorized into three antimicrobial resistance profiles: singular class, two classes, and three or more classes, and were distributed based on their biofilm formation ability (Table 4). Among multidrug-resistant *Staph. aureus* isolates (exhibiting resistance to three or more classes of antimicrobials), 83.3% (5/6) were categorized as strong biofilm producers, while 16.7% (1/6) belonged to the NP category (Table 4). Notably, the beta-lactam class was prevalent among all multidrug-resistant *Staph. aureus* isolates.

In the case of the 18 multidrug-resistant *Strep. uberis* isolates, 66.7% were classified as NP, 16.6% as strong biofilm producers, and another 16.6% as moderate biofilm producers (Table 4). The beta-lactam class was prominent among all multidrug-resistant *Strep. uberis* isolates.

Regarding *Strep. uberis* isolates categorized as strong biofilm producers, 15.8% (3/19) exhibited multiresistance. Isolates displaying moderate biofilm formation (10.3% or 3/29) and those categorized as non-biofilm producers (34.2% or 12/35) were also identified as multidrug-resistant. The most commonly associated classes of antimicrobials with multidrug resistance in *Strep. uberis* isolates were beta-lactams (100%) and tetracycline (94.73%). However, no significant association was found between the antimicrobial class causing multidrug resistance and biofilm formation.

## 4. Discussion

*Staph. aureus* and *Strep. uberis* are major causes of intramammary infections among dairy cows, with their capacity for biofilm formation recognized as a crucial virulence trait influencing mastitis pathogenesis and antimicrobial treatments. Our study assessed biofilm production and antimicrobial resistance in these isolates from mastitis-affected cows, which can help to understand the relationship between biofilm formation and antimicrobial resistance.

Our findings revealed that 57.36% of *Staph. aureus* isolates demonstrated biofilm production, consistent with previous reports (62.5%) [28]. However, notably high levels (54.8%) of *Staph. aureus* classified as NP were detected in Brazilian dairy herds [29]. This variance in biofilm-forming potential among *Staph. aureus* isolates in bovine mastitis highlight the potential chronicity of infections induced by this pathogen. Nonetheless, there remains a need to further investigate factors such as environmental stress, strain diversity, and location, which may influence gene expression related to biofilm production [30].

Discrepancies observed in biofilm evaluation might be attributed to variations in assay methodologies. While the microplate-based biofilm evaluation is considered the gold standard, its reliance on conditions influencing microbial growth and biofilm formation [31,32].

Biofilm formation often confers enhanced antimicrobial resistance [31,33]. Contrary to expectations, our study did not find a significant difference in antimicrobial susceptibility between biofilm and non-biofilm-producing *Staph. aureus* isolates. However, Rychshanova et al. [31] demonstrated that 69.4% of biofilm-producing *Staph. aureus* exhibited resistance to at least one antimicrobial class evaluated. The biofilm acts as a physical barrier, impeding direct contact between antimicrobials and microorganisms, a condition not mimicked during our in vitro testing. The lack of association between biofilm production and observed antimicrobial resistance might be explained by the absence of environmental conditions during our in vitro antimicrobial susceptibility tests. Additionally, the use of antimicrobials during the biofilm production evaluation can possibly improve the biofilm production capacity.

For *Strep. uberis*, 53.78% of the isolates demonstrated biofilm formation capacity. However, Dieser et al. [33] observed a higher percentage of isolates (87.5%) classified as weak or non-biofilm producers. Magagula [34] described that all the *Strep. uberis* isolates evaluated presented biofilm formation capacity, but only 17.8% (*n* = 30) were classified as strong biofilm producers. As previously mentioned, these variations in biofilm results can be attributed to variations in the strains evaluated, as well as in the expression of genes related to biofilm formation. Moliva et al. [35] demonstrated a correlation between the presence of virulence genes associated with the adhesion process (*gapC*, *hasABC*, *lbp*, *pauA*, and *sua*) and distinct biofilm formation patterns observed in *Strep. uberis*. Greeshma et al. [36] showed that isolates without *luxS* gene cannot produce a robust biofilm, leading to the inference that additional genes may be involved in regulating biofilm production. Alternatively, the luxS gene might play a regulatory role in one or more genes associated with biofilm formation in *Strep. uberis*.

Antimicrobial susceptibility testing revealed that over 90% of *Staph. aureus* isolates were susceptible to most of the evaluated antimicrobials. However, these isolates displayed lower sensitivity to ampicillin (49.2%), tetracycline (47.2%), and penicillin (7.1%). In line with our results, *Staph. aureus* isolates from Brazilian herds also exhibited reduced sensitivity to penicillin and ampicillin [37]. Kaczorek [38] described that *Staph. aureus* strains showed high resistance to penicillin (57%), oxytetracycline (25%) and tetracycline (18%). Among the isolates evaluated by Kaczorek, 70% of the isolates presented the ability to produce biofilms [38]. Additionally, the mastitis presentation form seemed to influence resistance patterns, with *Staph. aureus* from CM displaying heightened beta-lactam resistance [39].

The diminished susceptibility to beta-lactams might relate to the presence of resistance genes (e.g., *mecA*, *mecC*, and *blaz*) [40]. Penicillin-binding proteins (PBPs) are cell wall transpeptidases that catalyze the assembly of cell wall peptidoglycan. Modification on the pbps can improve the antimicrobial resistance to β-lactams due to the membrane proteins’ lower affinities to oxacillin and penicillin. Neelam et al. described that gene *mecA*, which is responsible for methicillin resistance, was detected in 23.64% (*n* = 13) of *Staph. aureus* isolates [40]. Aslantas et al. [28] demonstrated that penicillin and oxacillin-resistant *Staph. aureus* harbored *blaz* and *mecA* genes, respectively. For *Strep. uberis*, modifications in the pbp2x regions are associated with β-lactam resistance [41]. The presence of the E381K, Q554E, and G600E substitutions on pbpx was numerically associated with lower bacteriological cure rates following treatment with a β-lactam compared with a non-β-lactam intramammary therapy [41]. Molecular characterization analysis could further confirm these findings. However, this was beyond the scope of our study.

In our investigation, *Strep. uberis* exhibited substantial resistance to oxacillin (80.6%), penicillin (80.6%), tetracycline (37.8%), and pirlimycin (14.2%). This is consistent with varying reports in different regions, which showed high resistance rates to oxacillin in Switzerland (64.7%) and Korea (33.3%) [42]. The resistance rates to tetracycline differed notably, ranging from 27.1% to our finding of 37.8% [43]. As a result of a meta-analysis, it was determined that the highest levels of resistance for *Strep. uberis* were observed in gentamicin and tetracycline worldwide [22]. Regional or herd disparities, compounded by selective pressure from antimicrobial use, might account for such variations [44,45]. Extensive use of specific antimicrobial classes, like aminoglycosides, tetracyclines, and fluoroquinolones in Brazilian herds [46], can influence antimicrobial resistance patterns. For instance, the resistance of *Strep. uberis* to tetracyclines was linked to their prevalent use in Brazilian dairy herds [47].

Multidrug resistance, characterized by resistance to three or more antimicrobial classes [27], was less common in *Staph. aureus* (1.52%) but more prevalent in *Strep. uberis* (15.9%). The number of *Strep. uberis* that showed multidrug resistance were higher than reported to Magagula et al. (6.4%) [34]. These authors concluded that the low overall resistance must be linked to regional differences and prudent use of antimicrobials in the dairy industry [34]. For *Staph. aureus*, resistance to beta-lactams, particularly penicillin and cephalosporins, was consistent among all multidrug-resistant isolates. In contrast, among multidrug-resistant *Strep. uberis*, the most frequent resistance was observed against beta-lactams and tetracyclines.

## 5. Conclusions

Both *Staph. aureus* and *Strep. uberis* exhibited substantial biofilm formation abilities, despite the fact that no correlation was found between the mastitis presentation form (CM and SCM) and their biofilm-forming capacity. Additionally, most isolates from both species displayed resistance to penicillin, ampicillin, and tetracycline. Notably, no association was observed between biofilm formation ability and antimicrobial resistance.

## Figures and Tables

**Table 1:** Biofilm formation phenotype for *Staph. aureus* and *Strep. uberis* isolates from clinical and subclinical mastitis.

Biofilm Production	Mastitis	*Staph. aureus* (*n* = 197)			*Strep. uberis* (*n* = 119)		
		OD ^1^	*n*	%	OD	*n*	%
NP ^2^	SCM ^3^ + CM ^4^	0.020 (0.001–0.096)	84	42.6	0.008 (0.001–0.103)	55	46.2
	SCM	0.019 (0.001–0.071)	45	22.8	0.014 (0.001–0.103)	14	11.7
	CM	0.031 (0.001–0.096)	39	19.8	0.006 (0.001–0.038)	41	34.4
Weak	SCM + CM	0.029 (0.001–0.146)	33	16.7	0.035 (0.008–0.294)	13	10.9
	SCM	0.024 (0.008–0.146)	21	10.6	-	-	-
	CM	0.038 (0.001–0.121)	12	6.1	0.035 (0.008–0.294)	13	10.9
Moderate	SCM + CM	0.031 (0.004–0.163)	37	18.8	0.025 (0.001–0.173)	30	25.2
	SCM	0.028 (0.004–0.061)	26	13.9	-	-	-
	CM	0.037 (0.014–0.163)	11	5.58	0.025 (0.001–0.173)	30	25.2
Strong	SCM + CM	0.057 (0.004–0.275)	43	21.8	0.021 (0.001–0.185)	21	17.6
	SCM	0.044 (0.004–0.090)	19	9.6	0.005	1	0.8
	CM	0.062 (0.007–0.275)	24	12.2	0.030 (0.001–0.185)	20	16.8

^1^ OD: average value of optical density, ^2^ NP: non-producing, ^3^ SCM: subclinical mastitis, ^4^ CM: clinical mastitis.

**Table 2:** Frequency of in vitro susceptibility to antimicrobials of *Staph. aureus* and *Strep. uberis* isolated from bovine mastitis.

Antimicrobial	Antimicrobial Class	*Staph. aureus* (*n* = 197)						*Strep. uberis* (*n* = 119)					
		S ^1^		R ^2^		NA ^3^		S		R		NA	
		*n*	%	*n*	%	*n*	%	*n*	(%)	*n*	%	*n*	%
Ampicillin	Beta-lactam	97	49.2	100	50.8	-	-	101	84.9	11	9.2	7	5.9
Amoxicillin/clavulanic acid	Beta-lactam	194	98.5	3	1.5	-	-	112	94.1	1	0.8	6	5.0
Oxacillin	Beta-lactam	194	98.5	2	1.0	1	0.5	16	13.4	96	80.7	7	5.9
Penicillin	Beta-lactam	14	7.5	183	92.9	-	-	15	12.6	96	80.7	8	6.7
Cephalotin	Cephalosporin	192	97.5	4	2.0	1	0.5	112	94.1	1	0.8	6	5.0
Ceftiofur	Cephalosporin	157	79.7	40	20.3	-	-	108	90.8	4	3.4	7	5.9
Gentamicin	Aminoglycoside	194	98.5	1	0.5	2	1.0	111	93.3	2	1.7	6	5.0
Tetracycline	Tetracycline	93	47.2	104	52.8	-	-	67	56.3	45	37.8	7	5.9
Enrofloxacin	Fluoroquinolone	183	92.9	14	7.1	-	-	101	84.9	11	9.2	7	5.9
Pirlimycin	Lincosamide	196	99.5	1	0.5	-	-	95	79.8	17	14.3	7	5.9
Erythromycin	Macrolide	190	96.4	5	2.5	2	1.0	101	84.9	5	4.2	13	11.0
Penicillin/novobiocin	Penicillin/Novobiocin	197	100.0	0	0.0	-	-	111	93.3	2	1.7	6	5.0

^1^ S: susceptible, ^2^ R: resistant, ^3^ non-evaluated isolates: Isolates categorized as ‘NA’ were excluded from the in vitro antimicrobial evaluation due to contamination at the time of assay.

**Table 3:** Effect of biofilm formation capacity, type of mastitis (CM or SCM), and causative pathogen (*Staph. aureus*, *Strep. uberis*) on resistance and multiresistance to antimicrobials.

	*p*-Value				
Multiresistance	Biofilm ^1^	Mastitis ^2^	Bacteria ^3^	Bac*Bio ^4^	Mastitis*Bio ^5^
	0.817	0.923	0.016	0.162	0.345
Resistance					
Ampicillin	0.1050	0.0005	0.001	0.8611	0.5992
Amoxicillin/clavulanic acid	0.9913	0.3272	0.7557	0.7016	0.9425
Oxacillin	0.1686	0.0001	0.0001	0.4979	0.7898
Penicillin	0.9620	0.0137	0.2346	0.6844	0.7331
Cephalotin	0.4050	0.7397	0.3024	0.2738	0.2579
Ceftiofur	0.5474	0.9243	0.0002	0.6789	0.6829
Gentamicin	0.9828	0.9979	0.9694	0.9987	0.0001
Tetracycline	0.1731	0.0025	0.0001	0.2252	0.4063
Enrofloxacin	0.8468	0.3553	0.3942	0.9390	0.7553
Pirlimycin	0.9620	0.234	0.0137	0.6844	0.7331
Erythromycin	0.4611	0.9148	0.3243	0.9096	0.6133
Penicillin/novobiocin	0.8427	0.8971	0.9051	0.9662	0.9479

^1^ Biofilm: producing or non-producing response, ^2^ Mastitis: mastitis presentation form (SCM and CM), ^3^ Bacteria: Staph. aureus or Strep. uberis, ^4^ Bac*Bio: interaction between type of causative pathogen and biofilm, ^5^ Mastitis*Bio: interaction between mastitis presentation form (CM or SCM) and biofilm.

**Table 4:** Frequency of *Staph. aureus* and *Strep. uberis* isolates, distributed according to the number of different antimicrobial classes with resistance and biofilm formation capacity.

			Biofilm Categories							
Antimicrobial Classes ^1^			Strong		Moderate		Weak		NP ^2^	
	Bacteria	Mastitis	*n*	%	*n*	%	*n*	%	*n*	%
1	*Staph. aureus*	CM ^3^	18	9.1	8	4.1	7	3.5	33	16.7
		SCM ^4^	12	6.1	19	9.6	14	7.1	35	17.7
	*Strep. uberis*	CM	9	9.3	18	18.6	9	9.3	12	12.4
		SCM	-	-	-	-	-	-	-	-
2	*Staph. aureus*	CM	5	2.5	-	-	5	2.5	5	2.5
		SCM	5	2.5	7	3.5	6	3.0	5	2.5
	*Strep. uberis*	CM	7	7.2	8	8.2	1	1	11	11.3
		SCM	-	-	-	-	-	-	-	-
3+	*Staph. aureus*	CM	-	-	-	-	-	-	-	-
		SCM	5	2.5	-	-	-	-	1	0.5
	*Strep. uberis*	CM	3	3.1	3	3.1	-	-	12	12.4
		SCM	-	-	-	-	-	-	-	-

^1^ Number of antimicrobial classes whose isolates showed antimicrobial resistance, ^2^ NP: non-producing biofilm, ^3^ CM: clinical mastitis, ^4^ SCM: subclinical mastitis.

## Data Availability

The raw data from this study will be made available by the authors (corresponding author) upon the request of any qualified researcher.
